# An Assessment of Selected Molecular and Biochemical Markers of the Folate Pathway as Potential Risk Factors for Fetal Trisomy 21 during the First Trimester of Pregnancy in the Polish Population

**DOI:** 10.3390/jcm11051190

**Published:** 2022-02-23

**Authors:** Katarzyna Ziółkowska, Kinga Toboła-Wróbel, Marek Pietryga, Grażyna Kasprzak, Aleksander Jamsheer, Ewa Wysocka

**Affiliations:** 1 Department of Laboratory Diagnostics, Poznan University of Medical Sciences, 61-701 Poznan, Poland; katarzyna.ziolkowska@ump.edu.pl (K.Z.); grazyna.kasprzak@ump.edu.pl (G.K.); ewysocka@ump.edu.pl (E.W.); 2 Prenatal Diagnostic Centre, Gynaecology and Obstetrics Hospital, Poznan University of Medical Sciences, 61-701 Poznan, Poland; marekp2003@gmail.com; 3 Department of Obstetrics and Women’s Health, Gynaecology, Obstetrics and Gynaecological Oncology, Poznan University of Medical Sciences, 61-701 Poznan, Poland; 4 Department of Medical Genetics, Poznan University of Medical Sciences, 61-701 Poznan, Poland; jamsheer@wp.pl; 5 Centers for Medical Genetics GENESIS, 60-406 Poznan, Poland

**Keywords:** trisomy 21, folate pathway, gene polymorphism, PAPP-A, free β-hCG

## Abstract

Are the maternal gene variants *MTHFR:* c.665C>T, *MTHFR:* c.1286A>C, *MTR:* c.2756A>G, *MTRR:* c.66A>G, *RFC1:* c.80C>T and *TCN2:* c.776G>C and blood markers of the folate pathway important factors in assessing the risk of fetal trisomy 21 (fetal-T21)? Twenty pregnant women with a high risk and twenty with a low risk of fetal-T21 underwent prenatal examination. Selected gene variants and folate pathway markers and pregnancy-associated plasma protein A (PAPP-A) and free β-subunit of human chorionic gonadotropin β (free-β-hCG) multiple of the medians (MoMs) were determined. The distributions of the alternative alleles and genotypes of the gene variants did not differ between the studied groups. There was no relationship between PAPP-A and β-hCG MoM values and the presence of allele alternative genotype variants. The occurrence of alternative variants of the selected genes and concentrations of most of the studied folate pathway markers may not play a crucial role in the risk of fetal-T21 in pregnant women. However, the relationships between erythrocyte folate concentrations and the occurrence of alternative variants: c.665C>T *MTHFR* and c.776G>C *TCN2*, as well as the methylmalonic acid concentration and the occurrence of alternative variant c.776G>C *TCN2* in pregnant women with fetal-T21, encourage further research. So far, of the biochemical markers, maternal PAPP-A and β-hCG MoM values remain independent risk factors for fetal-T21.

## 1. Introduction

**Figure 1:**
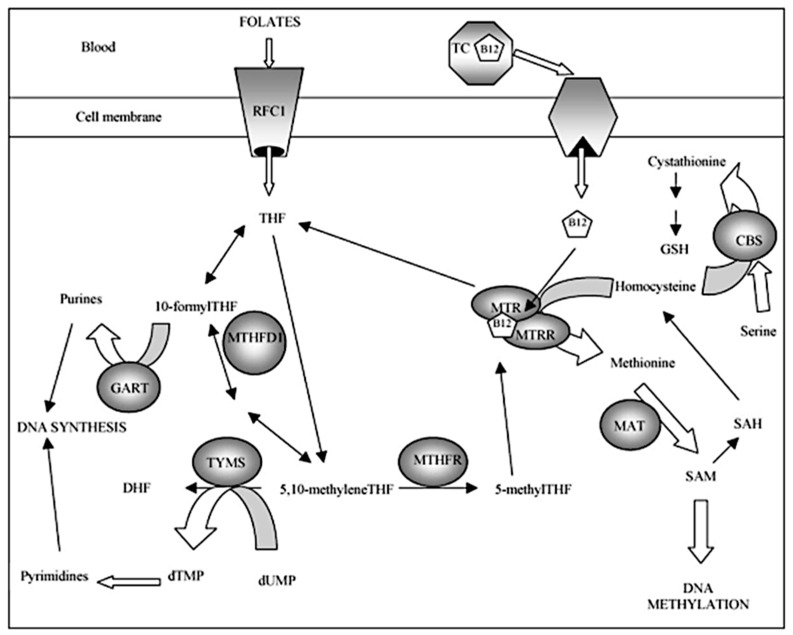
Simplified overview of the human folate metabolic pathway [10]. Enzymes: CBS: cystathionine b-synthase; GART: phosphoribosyl glycinamide transformylase; MTHFD1: methylenetetrahydrofolate dehydrogenase; MAT: methionine adenosyltransferase; MTHFR: methylenetetrahydrofolate reductase; MTR: methionine synthase; MTRR: methionine synthase reductase; RFC1: reduced folate carrier; TC (TCN2): transcobalamin; TYMS: thymidylate synthase. Metabolites: DHF: dihydrofolate; GSH: glutathione; THF: tetrahydrofolate; dTMP: deoxythymidine monophosphate; dUMP: deoxyuridine monophosphate; SAH: S-adenosyl homocysteine; SAM: S-adenosylmethionine. Cofactors: B12: vitamin B12 or cobalamin.

Trisomy 21 (T21), Down syndrome, is one of the most common prenatally diagnosed aneuploidies. Since the 1990s, there has been a main diagnostic scheme, implemented between 11 and 13 + 6 weeks of pregnancy, created by Professor Kypros Nicolaides, a world expert in fetal medicine. It consists of the assessment of the concentration of free β-subunit of human chorionic gonadotropin β (free β-hCG) and pregnancy-associated plasma protein A (PAPP-A) in the mother’s serum, with maternal age and measurement of nuchal translucency (NT) and the heart rate (FHR) in a fetus by ultrasound evaluation, which enable the detection of approximately 80–90% of T21 cases at false-positive rate results (FPR) of about 5% [1,2,3,4]. There are also other additional ultrasound markers to increase the sensitivity of prenatal screening, such as nasal bone (NB), tricuspid flow (TF) and ductus venosus (DV). However, scientists have long been looking for other risk factors and mechanisms leading to nondisjunction in meiotic division I or II, especially maternal, which results in the formation of a classic trisomy, most often trisomy 21 [5,6,7,8,9,10,11,12,13,14,15,16,17,18]. In the last 20 years, researchers have analyzed the impact of disorders in the process of folate metabolism depending on the occurrence of various gene variants in this pathway. The most biologically active form of folate, necessary for cellular transformations, is L-5-methylenetetrahydrofolate (L-5-MTHF). On the other hand, the reduced folate conveyor (RFC) enables the transport of folate to inside the cells. The active form of folate results from the reduction of 5,10-methylenetetrahydrofolate with the participation of the enzyme methylenetetrahydrofolate reductase (MTHFR) encoded by the *MTHFR* gene. Then, L-5-MTHF is involved in the remethylating of homocysteine to methionine catalyzed by methionine synthase (MTR) in the presence of the cofactor, vitamin B_12_ (Vit. B_12_). The resulting tetrahydrofolate (THF) is necessary to produce purines and pyrimidines, which are used to synthesize nucleic acids. Methionine synthase reductase (MTRR) is involved in maintaining the proper activity of MTR [5,6,7,8,9,10,11,12,13,14,15,16]. Vitamin B_12_ is an important cofactor involved in the remethylating of homocysteine to methionine. It is not synthesized in humans and must be supplied by foods or supplements. To obtain a biologically active form, Vit. B_12_ is bound to the transport protein holotranscobalamin II (TCN2), encoded by the *TCN2* gene. Via the *TCN2* protein, Vit. B_12_ is introduced within the cell and becomes metabolically available. Changes in the protein sequence of transcobalamin II may affect the ability of Vit. B_12_ to bind, or disrupt the binding of, the complex *TCN2*–Vit. B_12_ to the receptor and therefore reduce the concentration and lower the availability of active Vit. B_12_ as a cofactor in the remethylating of homocysteine to methionine [19,20,21,22]. Vitamin B_12_ is also a cofactor in the transformation of methylmalonyl-CoA into succinyl-CoA; hence, its deficiency may lead to an increase in the concentration of methylmalonic acid (MMA), which is an early indicator of this deficiency [22]. The next step in the folate transformation pathway is the conversion of methionine to S-adenosylmethionine (SAM), which is the main contributor of the methyl groups that is involved in the regulation of the functions of nucleic acids, mainly the methylation processes (Figure 1) [5,6,7,8,9,10,11,12,13,14,15,16].

Folate metabolism disorders may lead to the impaired synthesis of purines and pyrimidines, thus affecting the DNA structure. On the other hand, disturbances in methylation may cause the improper regulation of gene expression and restructuring of the chromatin structure, leading to anomalies in the formation of the spindle of the division and to disorders of the nondisjunction of or meiotic division I or II [5,6,7,8,9,10,11,12,13,14,16,17,18].

According to many authors, the abnormal segregation of chromosomes during gamete division that poses the risk of trisomy may be associated with the occurrence of gene variants of the folate pathway, in particular: c.665C>T *MTHFR* (rs1801131), c.1286A>C *MTHFR* (rs1801133), c.2756A>G *MTR* (rs1805087), c.66A>G *MTRR* (rs1801394), c.80C>T *RFC1* (rs1232681406) and c.776G>C *TCN2* (rs1801198), which, in turn, may lead to abnormal concentrations of biochemical parameters of this pathway [5,6,7,8,9,10,11,15,16,17,18,23,24,25,26,27,28,29,30].

We were motivated by these hypotheses to investigate whether the occurrence of alternative alleles and genotypes of selected gene variants of the folate pathway and changes in the concentrations of biochemical markers of this pathway (homocysteine, vitamin B_12_, methylmalonic acid and folic acid in the blood and serum) are important in the occurrence of T21 in maternal fetuses—carriers of these genetic variants in the Polish population. The values of PAPP-A and free β-hCG MoM are undeniable laboratory markers of the first trimester of pregnancy in the prenatal diagnosis of T21. This study also examined whether there are differences in the occurrence of reference and alternative variants of genes of the folate pathway in pregnant women carriers of these variants in whom abnormal values of PAPP-A and free β-hCG MoM characteristic for T21 were observed.

## 2. Aims

Are variants of the folate pathway genes c.665C>T *MTHFR* (rs1801131), c.1286A>C *MTHFR* (rs1801133), c.2756A>G *MTR* (rs1805087), c.66A>G *MTRR* (rs1801394), c.80C>T *RFC1* (rs1232681406) and c.776G>C *TCN2* (rs1801198) and concentrations of biochemical markers of this pathway (homocysteine, Vit. B_12_, MMA and folic acid in the blood and serum), as well as MoM values of PAPP-A and free subunit of β-hCG, contributors to the risk of T21 in the fetuses of Caucasian pregnant women from the Polish province of Greater Poland?

## 3. Materials and Methods

The study included 40 Caucasian women, residents of the Province of Greater Poland (Wielkopolska) and patients of the Prenatal Diagnostics Gynecology and Obstetrics Center of the Clinical Hospital of the Medical University in Poznań (between 2019 and January 2021), who were assessed for the risk of fetal chromosomal abnormalities between 11 and 13 + 6 weeks of pregnancy. The prenatal examination included an ultrasound assessment of the fetal anatomy and measurement of the concentration of PAPP-A and free β-hCG in the serum of the pregnant women.

Twenty pregnant women with a high risk of fetal T21 (≥1:300) who underwent genetic amniocentesis, in accordance with the recommendations of the Polish Society of Gynecologists and Obstetricians, and a karyotype result confirmed fetal T21. The control group consisted of 20 pregnant women with a low risk (<1:1000) of fetal T21.

The study group’s age (36 ± 6.0 year) and BMI (23.8 ± 3.5 kg/m^2^) did not differ from the control group’s age (38 ± 4.0) and BMI (24.4 ± 4.6).

The ultrasound examination was performed using the Voluson E8 ultrasound (General Electric Company (GE), Fairfield, CT, USA) and included the assessment of fetal anatomy with fetal heart rate (FHR) and measurement of nuchal translucency (NT) as a marker of the risk of chromosomal abnormalities (trisomy 21, 18 and 13), according to the standards of the Fetal Medicine Foundation and recommendations of the Ultrasound Section of the Polish Society of Gynecologists and Obstetricians [1,2,3,4].

During diagnosis in the first trimester of pregnancy, 7.5-mL blood serum from each pregnant women was taken for measurement of the concentrations of PAPP-A, free β-hCG, homocysteine (HCY), Vit. B_12_, serum folates (Folate _SER_, and MMA, and 2 mL of blood was taken with the anticoagulant ethylenediamine-tetraacetic acid (EDTA) to test for concentrations of folic acid in the red blood cells (Folate _RBC_). Additionally, 2 mL of blood was collected with EDTA for DNA isolation. The research project was approved by the Bioethics Committee of the Poznan University of Medical Sciences, Resolution No. 1006/18 of 11 October 2018, and the patients gave their written informed consent for performing the study procedures.

The concentrations of PAPP-A and free β-hCG in pregnant serum were assessed by the time-delayed fluorescence immunological method using the Kryptor Compact Plus device (B·R·A·H·M·S GmbH, Hennigsdorf, Germany).

Calculation of the risk of chromosomal aberrations considering biochemical and ultrasound markers was performed using the software © 2000–2016 Astraia (Astraia Software Gmbh, Munich, Germany) [1].

In patients with an increased risk of fetal-T21, amniocentesis was performed by collecting approximately 20 mL of amniotic fluid. The amniocytes from the amniotic fluid were cultured. After its completion and obtaining cytogenetic preparations, the chromosomes in the metaphase stage, stained by the G-line method, were analyzed to assess the karyotype [1].

The analysis of variants of the genes of the folate pathway was performed from DNA isolated from the blood of the pregnant women: c.665C>T *MTHFR*, c.1286A>C *MTHFR*, c.2756A>G *MTR*, c.66A>G *MTRR*, c.80C>T *RFC1* and c.776G>C *TCN2*. Some variants differed in nomenclature from those previously used in publications in compliance with the current guidelines for the nomenclature of single-nucleotide genetic variants of the Human Genome Variation Society (HGVS). The changes concerned: *MTHFR* (common nomenclature 677C>T), presented here as 665C>T (p.Ala222Val), and *MTHFR* (common nomenclature 1298A>C), presented here as 1286A>C (p.Glu429Ala) [20]. However, in the case of the variant c.776G>C *TCN2*, previously, the nomenclature c.776C>G *TCN2* was used, and now, it is *TCN2* -rs1801198- c.776G>C (p.Arg259Pro) NM_000355.4, due to the change of the reference sequence from C nucleotide to G nucleotide, despite the higher frequency of the C allele in the Caucasian population (61.7% with the C allele vs. 38.3% with the G allele, according to the GnomAD database). The authors of the publications cited in this paper used the previous nomenclature, and the more recent publications did not analyze the c.776G>C *TCN2* variant in relation to the risk of fetal T21 in mother carriers of this genetic variant. Our work is probably the first to present the above issue in pregnant women of the Polish population or even European in this way. In the study, alleles and genotypes were called “references” if they were considered references by the HGVS guidelines and human genetic databases. All other variants at the same genomic position were called “alternatives”. Usually, the “reference” variants occur more frequently in the Caucasian population than the “alternative” ones; however, exceptions to this rule exist. This current nomenclature may differ from the past nomenclature used by the authors of the cited publications, who used the terms “correct” or “reference” for alleles and genotypes occurring with greater frequency in the analyzed population and “mutated” for the less frequently occurring.

Polymorphic gene variants were analyzed by amplifying DNA fragments followed by sequencing using the modified Sanger method using the polymerase chain reaction (PCR). A sequential PCR reaction was performed using specific primers and the addition of dideoxynucleotides (ddNTPs). The sequences of the genetic variants were read by separating the terminated products of the PCR reaction on an ABI Genetic Analyzer 3130x1 sequencer, 50-cm capillary, POP7 polymer (Applied Biosystems Thermo Fisher Scientific, Waltham, MA, USA).

The electrochemiluminescence method (ECLIA) on the Cobas c601 analyzer (Hitachi/ROCHE Diagnostics Polska, ROCHE Holding AG, Basel, Switzerland) was used to determine the concentrations of Folate _SER_ and Folate _RBC_ and serum Vit. B_12_. Homocysteine was determined by the enzymatic method on the Cobas c501 analyzer (Hitachi/ROCHE Diagnostics, ROCHE Holding AG, Basel, Switzerland) and methylmalonic acid (MMA) was determined by the immunoenzymatically ELISA method, using the 800 TS Absorbance Reader (BioTek, Santa Clara, CA, USA). The following reference values were considered: Folate _SER_ 4.8–37.3 ng/mL, Folate _RBC_ 212.0–534.0 ng/mL, Vit. B_12_ 37.5–188.0 pg/mL, HCY 3.0–12.0 µmol/L and MMA 0.3–90.0 ng/mL.

Statistical analysis was performed with the MedCalc Statistical Software Version 19.8 software (Ostend, Belgium). The Kolmogorov–Smirnov test was used to assess the parametric distribution of the investigated quantitative features. To analyze the statistical significance of the obtained differences in the distribution of the quantitative features, we used the Student’s *t*-test for features with a parametric distribution (data presented as the mean ± standard deviation) and the Mann–Whitney rank test for features with a nonparametric distribution (data presented as the median and minimum–maximum). The relationships between the specific variant of the genotype and the concentration of the parameters in the blood serum of the patient were examined using a regression analysis. The distributions of the allele variants, as well as the reference and alternative genotypes within the studied genes, were analyzed using the chi-square test. The odds ratio (OR) of the occurrence of T21 in a fetus of a mother with a specific variant or combination of variants of the studied genes was assessed using the logistic regression test. A *p*-value of <0.05 was considered statistically significant.

## 4. Results

**Figure 2:**
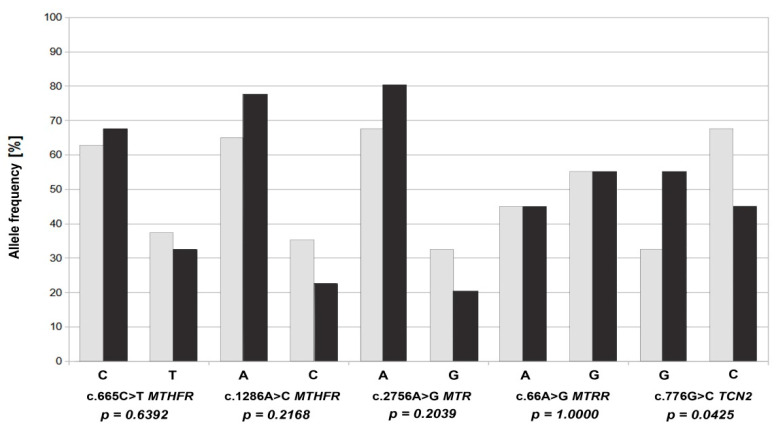
Frequency of occurrence of reference and alternative alleles of selected variants c.665C>T *MTHFR*, c.1286A>C *MTHFR*, c.2756A>G *MTR*, c.66A>G *MTRR* and c.776G>C *TCN2* in the control group (dark bar) and the study group (light bar).

The analysis of the frequency of the reference and alternative alleles (Figure 2) for the individual gene variants of the folate pathway showed that the highest percentage of alternative alleles in the study group occurred in the case of the c. 66A>G *MTRR* variant, constituting 55%, with a similar incidence in the control group (55%). In the case of the other variants, alternative alleles occurred more often in the control group than in the study group, with the highest percentage being 67% for the c.776G>C *TCN2* polymorphic variant (*p* = 0.042) (Table 1). For c.665C>T in *MTHFR*, the frequency of alternative alleles in the study group was 32.5%, and in the control group, 37.5%. In the case of the c.1286A>C *MTHFR* variant, the frequency of alternative alleles in the study and control groups was 22.5% and 35%, respectively. On the other hand, reference alleles were present in the study group with a frequency as high as 77.5%. Similarly, in the c.2756A>G *MTR* variant, the frequency of reference alleles in the study group was 80%, and the alternative alleles were only 20%. In the c.80C>T *RFC1* variant, only reference alleles were present in both groups.

**Figure 3:**
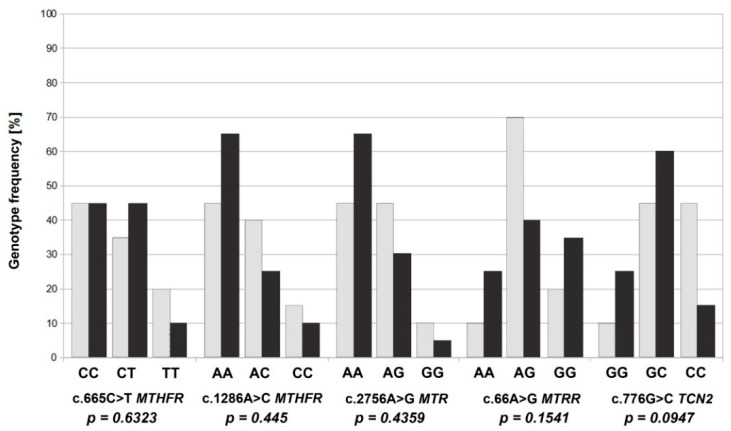
The frequency of the genotypes (homozygous and heterozygous) of selected variants c.665C>T *MTHFR*, c.1286A>C *MTHFR*, c.2756A>G *MTR*, c.66A>G *MTRR* and c.776G>C *TCN2* in the control group (dark bar) and the study group (light bar).

For an analysis of the frequency of the genotypes, we assessed alternative homozygotes and heterozygotes, as well as reference homozygotes. In the case of the c.665C>T *MTHFR* variant, the heterozygous CT was the most common (45%) in the study group. On the other hand, homozygous TT was more common (20%) in the control group. In the c.1286A>C *MTHFR* variant, the CC and AC genotypes occurred more often in the control group, 15% and 40%, respectively, while, in the study group, the frequencies were lower, at 10% and 25%, respectively. We saw similar results for the distribution of the genotypes in the c.2756A>G *MTR* variant. The highest percentage of GG homozygotes of the c.66A>G *MTRR* variant occurred in the study group (35%), while, in the control group, it was 20% (Figure 3). An analysis of the distribution of the genotypes in the individual genetic variants did not show any significant differences between the groups in terms of the presence of reference homozygotes and alternative homozygotes and heterozygotes (Table S1 Supplementary Data). An analysis of the frequency of combinations of the genotypes of linked variants of the genes of the folate pathway was also performed, and no statistically significant correlation was observed with the occurrence of trisomy 21 in fetuses of mothers carrying these systems (Table S2 Supplementary Data).

An analysis of the HCY, Vit. B_12_, Folate _SER_ and Folate _RBC_ concentrations showed the study group’s results within the reference values and did not differ from the concentrations that occurred in the control group. Statistically significant differences between the two groups were observed in the MMA values, where the mean value was 1.105 ng/mL in the study group and 6.013 ng/mL in the control group (Table 2). In the next stage, we checked whether concentrations of the biochemical markers, mother’s age and BMI differed in patient carriers of the reference genotypes and homo- and heterozygotes of alternative gene variants of the folate pathway, and a statistically significant correlation was found in the concentration of Folate _SER_ for the variant c.1286A>C *MTHFR*. There were also differences correlating with the age of the patients between carriers of homo- and heterozygous alternative genotypes and patients with the reference genotype in the variants c.665C>T *MTHFR* and c.2756A>G *MTR* (Table 3). An analysis by the multivariate regression model showed no correlation between the changes in the concentrations of the tested laboratory parameters and the presence of alternative alleles in each variant and, similarly for the alternative genotypes, both homo- and heterozygous for the analyzed variants of the genes of the folate pathway, in both groups of patients (Table 4 and Table S3 Supplementary Data). In the study group, there was a statistically significant correlation between the Folate _RBC_ concentration and the presence of hetero- and homozygous genotypes of alternative variants c.665C>T *MTHFR* and c.776G>C *TCN2* (*p* = 0.046) (Table 5). We tried to assess the relationships between the occurrence of alternative variants of the genotypes in a homozygous system (CC c.1286A>C *MTHFR*, TT c.665C>T *MTHFR*, GG c.2756A>G *MTR*, GG c.66A>G *MTRR* and CC c.776G>C *TCN2*) and the concentration of the studied biochemical parameters (HCY, Vit. B_12_, Folate _SER_, Folate _RBC_, MMA, PAPP-A MoM and free β-hCG MoM) using a multivariate regression model analysis. In the course of the statistical analyses, we did not observe any relationship when the control and study groups were considered together. Then, a small number of patients were presented with homozygous variants in the control and the study groups, separately. However, three females of the study group were found with a positive correlation of CC c.776G>C *TCN2* and MMA (*p* = 0.026), and four females of the control group were found with the positive correlation of GG c.66A>G *MTRR* and Folate _RBC_ (*p* = 0.043).

There was also no relationship between the age of the patients enrolled in the study and changes in the concentrations of biochemical parameters (Table S4 Supplementary Data).

In the study group (*n* = 20), the values characteristic for the risk of T21, i.e., PAPP-A < 0.500 MoM and free β-hCG > 2.00 MoM, were observed in six cases. Then, the presence of alternative genotypes of the studied gene variants was analyzed in this group (*n* = 6). The GC heterozygous genotype for the c.776G>C *TCN2* variant was present in five cases, and in three cases, the CT genotype was found for the c.665C>T variant in the *MTHFR*. For the remaining gene variants, no differences between the reference and alternative genotypes were observed (Table 6).

In further analyses, we assessed whether there were significant differences in the number of cumulated hetero- and homozygous genotypes of the studied variants, depending on the group of patients. It was observed that, for one patient in the control group, there was a statistically significantly greater number of genotype variants containing alternative alleles (Table 7). However, there was no correlation between the cumulative number of alternative genotypes, both hetero and homozygous for the tested variants, and changes in the biochemical parameters (Table 8).

In the last stage, it was checked whether the presence of alternative alleles or a combination of genotypes of the studied gene variants of the folic acid pathway in pregnant women may have an impact on the risk of fetal T21. In the case of the c.665C>T *MTHFR* variant, in homozygous and heterozygous carriers of alternative alleles in the following systems: CT vs. CC and TT + CT vs. CC, where the OR was 1.446 and 1.5195, respectively, fetal T21 was present. However, this analysis was not statistically significant (*p* = 0.598 and *p* = 0.519). A similar situation was observed in the case of the c.66A>G *MTRR* polymorphic variant; homozygous carriers of the alternative alleles had a fetus with trisomy 21. However, statistical significance was obtained only in the c.776G>C *TCN2* variant, where the alternative C allele and the CC homozygous genotype were more frequent in pregnant women in the control group (Table 9).

## 5. Discussion

In the last 20 years, researchers have shown interest in gene variants of the folic acid pathway and their impact on the impaired synthesis of nucleic acid precursors and disorders of DNA methylation, which could lead to nondisjunction in and meiotic division I and II [5,6,7,8,9,10,11,16,17,18]. This paper presented selected variants of genes that may affect the activity of enzymes involved in the folate cycle. The analysis of the selected variants did not show a statistically significant relationship between the occurrence of alternative alleles and genotypes in a group of Caucasian pregnant women and Wielkopolska pregnant women with fetal trisomy 21, unlike the results of other authors [5,6,7,8,10,11,15,18,24,27]. Our analysis of the combinations of genotypes in alternative genes did not show differences between the groups and did not confirm the higher frequency of alternative alleles and genotypes in the fetal T21 group. However, it was observed that, in the case of the c.665C>T *MTHFR* variant in homozygous and heterozygous carriers of alternative alleles in the following systems: CT vs. CC and TT + CT vs. CC (OR:1.446 and 1.5195) and the c.66A>G *MTRR* variant in homozygous carriers GG vs. AG + AA genotype (OR:2.153), fetal T21 was more frequent, similar to other studies [5,7,16,18,24,25,26,27]. Our results were not statistically significant; however, this issue also occurred in other studies [17,25,28,29,30]. Some authors observed that, in a population of white American, Chinese and Italian women, the combination of the CT and TT genotypes of the c.665C>T *MTHFR* variant and the GG genotype for the c.66A>G variant in the *MTRR* (OR = 6.0, *p* < 0.025; Wang) significantly increased the risk for trisomy 21 in fetuses of women carriers of these alternative genotypes [2,4,21]. The lack of statistical significance in our research may depend on the size of the groups and the studied population, because in the Polish population, there are no significant differences in the frequency of the alleles and genotypes of the studied alternative variants between women with fetal trisomy 21 and without the abnormality. Other authors observed differences between groups that probably arose from higher frequencies of alternative genotypes of the folate pathway gene variants in their study populations of mothers with trisomy 21 in their fetuses. This phenomenon mainly occurred in American, Chinese, Indian and Italian populations [5,6,7,10,11,15,18,24,26,27]. Sukla et al., in a study of a group of more than 250 Indian women with the c.776C>G TCN2 variant, discovered that the risk of Down syndrome in children of carriers of alternative genotypes was higher (then referred to as “mutated”). In the works of Fintelman-Rotriques et al. and Zampieri et al., studying a group of more than 200 Brazilian pregnant women with fetal trisomy 21, no correlation of alternative genotypes of the c.776G>C TCN2 variant with the risk of T21 in children carrying these genotypes was found [9,28]. Similar results were observed in this study. In studies of the Polish population, other authors have also failed to obtain results confirming the increase in the frequency of alternative alleles and genotypes in groups of Polish pregnant women with trisomy 21 in fetuses [14]. Some authors pointed to differences in serum HCY concentrations in the groups of pregnant women with fetal trisomy 21 correlating with the presence of homozygous and heterozygous mutant genotypes of the c.665C>T *MTHFR* polymorphism, especially the TT genotype [7,10,26]. Our analysis of the HCY, Vit. B_12_, Folate _SER_ and Folate _RBC_ concentrations showed no statistically significant differences in the studied groups in relation to the presence of homo- and heterozygous genotypes of the selected variants. There was also no relationship between the age of the patients enrolled in the study and changes in the concentrations of the biochemical parameters. Coppede et al. observed a relationship between a decrease in the Folate _SER_ concentration and an increase in the HCY concentration with the occurrence of the TT genotype of the c.665C>T *MTHFR* variant [10]. In our analysis, in the study group, there was a statistically significant relationship between the concentration of Folate _RBC_ and the presence of hetero- and homozygous genotypes of alternative variants c.665C>T *MTHFR* and c.776G>C *TCN2* and a relation between the MMA concentration and the occurrence of alternative genotypes GG for variant c. 2756A>G *MTR* and CC for variant c.776G>C *TCN2*. The concentration of MMA differed significantly between the groups and was lower in the patients with fetal T21. However, this was not consistent with the hypothesis that higher MMA values may indicate a decrease in the concentration of active vitamin B_12_ and, thus, a disturbance in the conversion of homocysteine to methionine, which, consequently, could lead to an increased risk of fetal T21. It should be remembered that the MMA concentrations in both groups were within the reference range and the doses of folate and Vit. B_12_ taken by pregnant women were not evaluated, so it is difficult to assess the observed differences. The values of PAPP-A MoM and free β-hCG MoM were characteristic of the risk of T21. These parameters were necessary for the screening (double test) to determine the risk of T21 in the fetus and to select the study group. It was also decided to evaluate these parameters in relation to the frequency of alternative genotypes of particular variants of the studied genes, but the analysis did not show a statistically significant relationship. However, in the cases of abnormal PAPP-A (<0.5 MoM) and β-hCG (>2.0 MoM) values, the most common was the heterozygous GC genotype of the c.776G>C *TCN2* variant and, additionally, the CT 665C>T *MTHFR* genotype. The small size of our study group made it impossible to draw conclusions about the validity of combining the changes in the parameters of the double test with the occurrence of gene variants leading to disorders of folate transformation; this issue requires further research. The discussion of researchers, which has been going on for over 20 years on the advisability of combining biochemical, ultrasound and molecular parameters, continues to guide diagnostics in the search for new parameters or modifications already used in order to be even more effective in detecting chromosome aberrations in the early weeks of pregnancy. However, the method of direct evaluation of free fetal DNA in the mother’s blood serum remains the most effective. The introduction of NIPT diagnostics, i.e., the analysis of free fetal DNA in the maternal circulation, is already routinely used in many countries. However, in Poland, the high cost of these tests means that this diagnostic is still not available to all pregnant women. Therefore, it remains reasonable to continue searching for and using the available research technologies in the field of noninvasive prenatal diagnostics, which will reduce the number of amniocentesis and increase the effectiveness in detecting chromosomal aberrations in fetuses.

## 6. Conclusions

It seems that the occurrence of alternative alleles and genotypes of selected variants of the genes of the folate pathway, as well as their relationship with the concentrations of most biochemical markers of this pathway, are not significant for the risk of T21 in the fetuses of pregnant women who are carriers of these variants.

The relationship between the Folate _RBC_ concentration and the presence of hetero- and homozygous genotypes of alternative variants c.665C>T *MTHFR* and c.776G>C *TCN2* and the concentration of MMA with the occurrence of alternative genotypes GG variant c.2756A>G *MTR* and CC variant c.776G>C *TCN2* in the group of women with fetal T21 encourages further research.

Maternal PAAP-A and β-hCG MoM values are independent risk factors for fetal T21.

## Figures and Tables

**Table 1:** Distribution of reference and alternative alleles in the control and study groups.

Groups	Allele	*N*	*p*-Value
c.665C>T *MTHFR* (rs1801131)			
Study	C	27	0.639
Study	T	13	
Control	C	25	
Control	T	15	
c.1286A>C *MTHFR* (rs1801133)			
Study	A	31	0.217
Study	C	9	
Control	A	26	
Control	C	14	
c.2756A>G *MTR* (rs1805087)			
Study	A	32	0.204
Study	G	8	
Control	A	27	
Control	G	13	
c.66A>G *MTRR* (rs1801394)			
Study	A	18	1.000
Study	G	22	
Control	A	18	
Control	G	22	
c.776G>C *TCN2* (rs1801198)			
Study	G	29	0.042
Study	C	11	
Control	G	13	
Control	C	27	

**Table 2:** Characteristics of the clinical and biochemical parameters in the control and study groups.

Parameters	Control Group	Study Group	*p*-Value
Patient’s age (years)	38 ± 4	36 ± 6	0.3062
BMI (kg/m^2^)	24.4 ± 4.6	23.8 ± 3.5	0.6263
HCY (µmol/L)	6.176 ± 1.378	7.137 ± 2.360	0.126
Vit. B_12_ (pg/mL)	57.434 ± 15.632	49.203 ± 14.335	0.0907
Folate _SER_ (ng/mL)	15.075 (6.3–26.9)	12.725 (5.49–62.6)	0.0818
Folate _RBC_ (ng/mL)	319.545 ± 46.518	358.31 ± 86.228	>0.05
MMA (ng/mL)	6.013 (0.56–34.98)	1.105 (0.188–80.113)	<0.0001
PAPP-A MoM	1.14 (0.41–1.98)	0.336 (1.32–1.857)	0.019
free β-hCG MoM	1.1 (0.46–2.51)	2.64 (0.59–9.76)	0.0029

HCY: homocysteine; Vit. B_12_: Vitamin B_12_; Folate _SER_: serum folate; Folate _RBC_: red blood cell folate, MMA: methylmalonic acid; PAPP-A MoM: median PAPP-A; free β-hCG MoM: median of the free β-hCG subunit.

**Table 3:** Analysis of the changes in the clinical and biochemical parameters depending on the presence of reference homozygous genotypes, as well as heterozygous and heterozygous genotypes of the studied variants of the genes of the folate pathway.

Test Parameters	Genetic Variants of the Folate Pathway		*p*-Value
	Reference Homozygote	Hetero- and Homozygous Alternative	
	665C>T *MTHFR*		
	CC (*n* = 18)	CT + TT (*n* = 22)	
Age (years)	35 ± 6	39 ± 3	0.0103
BMI (kg/m^2^)	24.1908 ± 3.8361	24.0482 ± 4.2881	0.9133
HCY (µmol/L)	6.6311 ± 2.1191	6.6782 ± 1.8876	0.9412
Vit. B_12_ (pg/mL)	51.5600 ± 13.6861	54.7586 ± 16.8176	0.5199
Folate _SER_ (ng/mL)	18.3706 ± 12.9428	12.9659 ± 5.0261	0.0792
Folate _RBC_ (ng/mL)	355.3 ± 93.1147	325.5318 ± 41.9387	0.1867
MMA (ng/mL)	2.9700 (0.188–34.98)	2.5415 (0.54–80.113)	0.7442
PAPPA MoM	0.7481 ± 0.5053	0.9019 ± 0.5985	0.3919
free β-hCG MoM	2.5843 ± 2.8672	2.1161 ± 1.7018	0.5252
	1286A>C *MTHFR*		
	AA (*n* = 22)	AC + CC (*n* = 18)	
Age (years)	38 ± 6	36 ± 5	0.4193
BMI (kg/m^2^)	24.4863 ± 24.4863	23.6553 ± 3.9584	0.5245
HCY (µmol/L)	6.7941 ± 2.1459	6.4894 ± 1.7751	0.6326
Vit. B_12_ (pg/mL)	50.5186 ± 15.1741	56.7422 ± 15.3513	0.2070
Folate _SER_ (ng/mL)	12.41 (5.49–62.6)	15.3450 (7.77–26.9)	0.0240
Folate _RBC_ (ng/mL)	335.3318 ± 51.3728	343.3222 ± 89.7874	0.7258
MMA (ng/mL)	1.8965 (0.401–80.113)	6.8645 (0.188–34.98)	0.3994
PAPPA MoM	0.9259 ± 0.6447	0.7189 ± 0.4163	0.2474
free β-hCG MoM	2.1468 ± 1.7383	2.5468 ± 2.8461	0.5875
	c.2756A>G *MTR*		
	AA (*n* = 22)	AG + GG (*n* = 18)	
Age (years)	36 ± 6	39 ± 4	0.0478
BMI (kg/m^2^)	23.9457 ± 3.9884	24.3160 ± 4.2084	0.7772
HCY (µmol/L)	6.2777 ± 1.9589	7.1206 ± 1.9338	0.1814
Vit. B_12_ (pg/mL)	51.7832 ± 16.2295	55.1967 ± 14.5083	0.4921
Folate _SER_ (ng/mL)	16.0636 ± 12.1031	14.5844 ± 5.7885	0.6374
Folate _RBC_ (ng/mL)	344.6273 ± 81.3418	331.9611 ± 5.6091	0.5778
MMA (ng/mL)	6.1350 (0.188–80.113)	1.0695 (0.401–60.816)	0.1656
PAPPA MoM	0.857 ± 0.591	0.8031 ± 0.5278	0.7652
free β-hCG MoM	2.6648 ± 2.583	1.9137 ± 1.8318	0.3060
	c.66A>G *MTRR*		
	AA (*n* = 7)	AG + GG (*n* = 33)	
Age (years)	35 ± 6	37 ± 5	0.3695
BMI (kg/m^2^)	22.676 ± 2.6449	24.4170 ± 4.2464	0.3065
HCY (µmol/L)	6.5586 ± 1.9924	6.6779 ± 1.9944	0.8864
Vit. B_12_ (pg/mL)	50.4229 ± 18.5376	53.9336 ± 14.8871	0.5899
Folate _SER_ (ng/mL)	13.68 (7.32–26.76)	14.00 (5.49–62.6)	0.9291
Folate _RBC_ (ng/mL)	373.5857 ± 113.02	331.5758 ± 57.6266	0.1538
MMA (ng/mL)	6.56 (0.54–34.93)	2.72 (0.188–80.113)	0.7086
PAPPA MoM	0.5631 ± 0.4976	0.8899 ± 0.5589	0.1613
free β-hCG MoM	3.0783 ± 3.1022	2.1674 ± 2.0932	0.3436
	c.776 C>G *TCN2*		
	CC (*n* = 12)	CG + GG (*n* = 28)	
Age (years)	39 ± 4	36 ± 5	0.0789
BMI (kg/m^2^)	23.6184 ± 4.3182	24.3241 ± 3.9778	0.6190
HCY (µmol/L)	6.6708 ± 2.0831	6.6511 ± 1.9574	0.9772
Vit. B_12_ (pg/mL)	54.2075 ± 10.8508	52.9386 ± 17.1231	0.8145
Folate _SER_ (ng/mL)	12.465 (7.57–26.9)	13.84 (5.49–62.6)	0.9294
Folate _RBC_ (ng/mL)	337.2167 ± 53.5840	339.6607 ± 77.3331	0.9214
MMA (ng/mL)	2.0350 (0.188–80.113)	4.0950 (0.401–60.816)	0.5354
PAPPA MoM	1.0306 ± 0.6020	0.7479 ± 0.52949	0.1434
free β-hCG MoM	1.4342 ± 0.8299	2.7593 ± 2.5928	0.1058

HCY: homocysteine; Vit. B_12_: Vitamin B_12_; Folate _SER_: serum folate; Folate _RBC_: red blood cell folate, MMA: methylmalonic acid; PAPP-A MoM: median PAPP-A; free β-hCG MoM: median of the free β-hCG subunit.

**Table 4:** Assessment of the relationship between the occurrence of alternative alleles in both hetero- and homozygous systems with the concentrations of the studied biochemical parameters based on the analysis of the multivariate regression model.

Parameters of Control and Study Groups	c.1286A>C *MTHFR*		c.665C>T *MTHFR*		c.2756A>G *MTR*		c.66A>G *MTRR*		c.776G>C *TCN2*	
	Allele C (*n* = 23)		Allele T (*n* = 28)		Allele G (*n* = 21)		Allele G (*n* = 44)		Allele C (*n* = 38)	
	Coefficient	*p*-Value	Coefficient	*p*-Value	Coefficient	*p*-Value	Coefficient	*p*-Value	Coefficient	*p*-Value
HCY (µmol/L)	−0.2074	0.6933	−0.1083	0.8407	0.6447	0.2423	−0.01039	0.9826	0.1744	0.7329
Vit. B_12_ (pg/mL)	4.4357	0.2784	−1.0703	0.7979	3.1238	0.4639	1.4485	0.6948	−0.4202	0.9155
Folate _RBC_ (ng/mL)	−5.7469	0.7563	−33.782	0.0782	−8.1303	0.6743	2.1095	0.8998	21.6294	0.2317
Folate _SER_ (ng/mL)	0.9647	0.7065	−4.1179	0.1198	0.2742	0.9183	−0.6883	0.7665	1.243	0.6179
MMA (ng/mL)	−2.2179	0.6327	−0.4493	0.9247	−0.08866	0.9854	0.8206	0.845	2.6185	0.5617
PAPP-A MoM	−0.1469	0.3299	0.06644	0.6627	0.005361	0.9724	0.0173	0.8988	0.1469	0.3148
free β-hCG MoM	0.0785	0.8982	−0.04186	0.9463	−0.5617	0.3762	0.3226	0.5619	−0.9328	0.12

HCY: homocysteine; Vit. B_12_: Vitamin B_12_; Folate _SER_: serum folate; Folate _RBC_: red blood cell folate, MMA: methylmalonic acid; PAPP-A MoM: median PAPP-A; free β-hCG MoM: median of the free β-hCG subunit.

**Table 5:** Assessment of the relationship between the occurrence of alternative genotype variants in both hetero- and homozygous systems with the concentrations of the tested biochemical parameters in the study group based on a multivariate regression model analysis.

Parameters of the Study Group	c.1286A>C *MTHFR*		c.665C>T *MTHFR*		c.2756A>G *MTR*		c.66A>G *MTRR*		c.776G>C *TCN2*	
	AC + CC (*n* = 7)		CT + TT (*n* = 11)		AG + GG (*n* = 7)		AG + GG (*n* = 15)		GC + CC (*n* = 15)	
	Coefficient	*p*-Value	Coefficient	*p*-Value	Coefficient	*p*-Value	Coefficient	*p*-Value	Coefficient	*p*-Value
free β-hCG MoM	2.1143	0.2272	−0.2669	0.8763	−0.9782	0.5254	−1.113	0.5501	−1.1337	0.5389
PAPP-A MoM	−0.08848	0.7391	0.2388	0.3788	−0.2188	0.3663	0.1623	0.5758	−0.2902	0.3191
MMA (ng/mL)	−1.1728	0.929	12.1823	0.3596	−6.9839	0.5542	13.0815	0.3485	11.9	0.4062
Folate _RBC_ (ng/mL)	−35.5647	0.3331	−104.3238	0.0105	−20.4323	0.5289	−42.7235	0.2679	128.4654	0.0046
Folate _SER_ (ng/mL)	−4.4338	0.5391	−11.5471	0.1217	−3.7273	0.5623	1.5842	0.8324	8.9313	0.2579
HCY (µmol/L)	0.06287	0.9637	−1.0299	0.4589	1.3878	0.2712	0.8271	0.5689	2.213	0.152
Vit. B_12_ (pg/mL)	0.01619	0.9984	11.2406	0.1781	9.1704	0.2176	1.8014	0.8316	−6.6602	0.4497

Free β-hCG MoM: median of the free β-hCG subunit; PAPP-A MoM: median PAPP-A; MMA: methylmalonic acid; Folate _RBC_: red blood cell folate; Folate _SER_: serum folate; HCY: homocysteine; Vit. B_12_: Vitamin B_12_.

**Table 6:** Distribution of individual gene variants of the folate pathway in patients with abnormal PAPP-A MoM results and trisomy 21 in the fetus.

Patient with T21 in the Fetus	c.665C>T *MTHFR*	c.1286A>C *MTHFR*	c.2756A>G *MTR*	c.66A>G *MTRR*	c.776G>C *TCN2*	Number of Alternate Genotypic Variants	PAPP-A MoM	β-hCG MoM	Age	BMI
No. 1	CC	CC	AG	AG	GG	3	0.31	3.74	37	23
No. 2	CT	AC	AG	AG	GC	5	0.28	8.37	36	20
No. 3	TT	AA	AG	AA	GC	3	0.42	3.25	36	23
No. 4	CT	AA	AA	AA	GC	2	0.13	3.2	36	25
No. 5	CT	AA	AA	GG	GC	3	0.3	3.63	39	27
No. 6	CC	AC	AA	AA	GC	2	0.34	9.76	32	18

**Table 7:** Comparison of the number of genotypes containing alternative allele variants in the studied groups of patients.

Group	Number of Alternate Genotypes. Median (Min–Max.)	*p*-Value
Study	3 (1–5)	0.0215
Control	4 (2–4)	

**Table 8:** Assessment of the relationship between changes in the concentrations of the tested biochemical parameters and the number of alternative genotypes of the variants of the studied genes in both the hetero- and homozygous systems based on a regression model analysis.

Parameters	Number of Alternate Genotypes	
	Coefficient	*p*-Value
HCY (µmol/L)	0.3119	0.3513
Vit. B_12_ (pg/mL)	4.2411	0.1005
Folate _SER_ (ng/mL)	−11.843	0.3216
Folate _RBC_ (ng/mL)	−0.9576	0.5625
MMA (ng/mL)	1.0863	0.7121
PAPPA MoM	0.03181	0.7437
free β-hCG MoM	−0.6527	0.0976

HCY: homocysteine; Vit. B_12_: Vitamin B_12_; Folate _SER_: serum folate; Folate _RBC_: red blood cell folate, MMA: methylmalonic acid; PAPP-A MoM: median PAPP-A; free β-hCG MoM: median of the free β-hCG subunit.

**Table 9:** Genetic association studies of the folate pathway genes as maternal risk factors for having a child with Down syndrome.

Variants of the Studied Genes	The Arrangement of Alleles and Genotypes in the Analyzed Variants	OR	(95% CI)	*p*-Value
c.665C>T *MTHFR*	T vs. C	OR = 0.7143	95% CI = 0.2815−1.8125	*p* = 0.4788
	TT vs. CC	OR = 0.5625	95% CI = 0.0803−3.9392	*p* = 0.5623
	CT vs. CC	OR = 1.4464	95% CI = 0.3668−5.7042	*p* = 0.598
	TT + CT vs. CC	OR = 1.5195	95% CI = 0.4255−5.4266	*p* = 0.519
	TT vs. CT + CC	OR = 0.4444	95% CI = 0.0716−2.7599	*p* = 0.3841
c.1286A>C *MTHFR*	C vs. A.	OR = 0.5392	95% CI = 0.2011−1.4458	*p* = 0.2196
	CC vs. AA	OR = 0.4615	95% CI = 0.0637−3.3456	*p* = 0.4442
	AC vs. AA	OR = 0.4327	95% CI = 0.1063−1.7615	*p* = 0.2422
	CC + AC vs. AA	OR = 0.4406	95% CI = 0.1234−1.5734	*p* = 0.2069
	CC vs. AC + AA	OR = 0.6296	95% CI = 0.0934−4.2437	*p* = 0.6346
c.2756A>G *MTR*	G vs. A	OR = 0.5192	95% CI = 0.1874−1.4383	*p* = 0.2074
	GG vs. AA	OR = 0.3462	95% CI = 0.0271−4.4178	*p* = 0.4142
	AG vs. AA	OR = 0.4615	95% CI = 0.1211−1.7586	*p* = 0.2573
	CC + AC vs. AA	OR = 0.4406	95% CI = 0.1234−1.5734	*p* = 0.2069
	CC vs. AC + AA	OR = 0.4737	95% CI = 0.0394−5.6879	*p* = 0.5557
c.66A>G *MTRR*	G vs. A.	OR = 1.000	95% CI = 0.4144−2.4132	*p* = 1.000
	GG vs. AG	OR = 4.375	95% CI = 0.8817−21.7077	*p* = 0.0709
	GG vs. AA	OR = 0.700	95% CI = 0.0902−5.4320	*p* = 0.7330
	AG vs. AA	OR = 0.2286	95% CI = 0.0357−1.4620	*p* = 0.119
	GG + AG vs. AA	OR = 0.3333	95% CI = 0.0564−1.9712.	*p* = 0.2257
	GG vs. AG + AA	OR = 2.1538	95% CI = 0.5155−9.0000	*p* = 0.293
c.776G>C *TCN2*	C vs. G.	OR = 0.3939	95% CI = 0.1588−0.9775	*p* = 0.0445
	CC vs. GC	OR = 0.25	95% CI = 0.522−1.1976	*p* = 0.0829
	CC vs. GG	OR = 0.1333	95% CI = 0.0164−1.0853	*p* = 0.0596
	GC vs. GG	OR = 0.5333	95%CI = 0.0836−3.4044	*p* = 0.5063
	CC + GC vs. GG	OR = 0.3333	95% CI = 0.0564−1.9712	*p* = 0.2257
	CC vs. GC + GG	OR = 0.2157	95% CI = 0.0476−0.9772	*p* = 0.0466
c.665C>T *MTHFR* + c.66A>G *MTRR*	CT + GG	OR = 3.3529	95% CI = 0.3179−35.3658	*p* = 0.3142

## Data Availability

The data contained within the article and Supplementary Materials are available on request from the authors.

## Supplementary Materials

The following supporting information can be downloaded at: https://www.mdpi.com/article/10.3390/jcm11051190/s1, Table S1. The distribution of genotypes of individual variants of genes of the folate pathway in the control and study groups. Table S2. Analysis of the frequency of combinations of genotypes of gene variants linked to changes in the folate pathway in the study and control groups. x = no combination /arrangement of genotypes observed. Table S3. Assessment of the relationship between the occurrence of alternative genotype variants in both hetero- and homozygous systems with the concentration of the tested biochemical parameters: (A) in all patients and (B) in the con-trol group, based on multivariate regression model analysis. (The study group is in the main manuscript). Table S4. Analysis of the correlation between the concentrations of the biochemical parameters and the age of the patients qualified for the study based on Spearman’s .correlation coefficient.
