# Supplementary material for: An Assessment of Selected Molecular and Biochemical Markers of the Folate Pathway as Potential Risk Factors for Fetal Trisomy 21 during the First Trimester of Pregnancy in the Polish Population
Source: J Clin Med. 2022 Feb 23;11(5):1190. doi: 10.3390/jcm11051190 (PMC8911130; doi:10.3390/jcm11051190)
Supplement: Supplementary file 1 [file jcm-11-01190-s001.zip › jcm-1511749-supplementary.pdf]

**Table S1.** The distribution of genotypes of individual variants of genes of the folate pathway in the control and study groups.

| Groups | Allele | N | p-value |
|---|---|---|---|
| **c.665C>T *MTHFR* (rs1801131)** | | | |
| Study | TT | 2 | 0.632 |
| Study | CC | 9 | |
| Study | CT | 9 | |
| Control | TT | 4 | |
| Control | CC | 9 | |
| Control | CT | 7 | |
| **c.1286A>C *MTHFR* (rs1801133)** | | | |
| Study | AA | 13 | 0.445 |
| Study | CC | 2 | |
| Study | AC | 5 | |
| Control | AA | 9 | |
| Control | CC | 3 | |
| Control | AC | 8 | |
| **c.2756A>G *MTR* (rs1805087)** | | | |
| Study | AA | 13 | 0 .436 |
| Study | GG | 1 | |
| Study | AG | 6 | |
| Control | AA | 9 | |
| Control | GG | 2 | |
| Control | AG | 9 | |
| **c.66A>G *MTRR* (rs1801394)** | | | |
| Study | AA | 5 | 0.154 |
| Study | GG | 7 | |
| Study | AG | 8 | |
| Control | AA | 2 | |
| Control | GG | 4 | |
| Control | AG | 14 | |
| **c.776G>C *TCN2* (rs1801198)** | | | |
| Study | CC | 3 | 0.095 |
| Study | GG | 5 | |
| Study | CG | 12 | |
| Control | CC | 9 | |
| Control | GG | 2 | |
| Control | CG | 9 | |

**Table S2.** Analysis of the frequency of combinations of genotypes of gene variants linked to changes in the folate pathway in the study and control groups. x = no combination /arrangement of genotypes observed.

| c.665C>T *MTHFR* / c.1286A>C *MTHFR* | | |
|---|---|---|
| **Combinations of genotypes** | **Study group** | Control group |
| **CC/AA** | 4 | 1 |
| **CC/AC** | 3 | 5 |
| **CC/CC** | 2 | 3 |
| **CT/AA** | 7 | 4 |
| **CT/AC** | 2 | 3 |
| **CT/CC** | x | x |
| **TT/AA** | 2 | 4 |
| **TT/AC** | x | x |
| **TT/CC** | x | x |
| **c.665C>T *MTHFR* / c.2756A>G *MTR*** | | |
| **Combinations of genotypes** | **Study group** | **Control group** |
| **CC/AA** | 7 | 3 |
| **CC/AG** | 2 | 6 |
| **CC/GG** | x | x |
| **CT/AA** | 5 | 5 |
| **CT/AG** | 3 | 1 |
| **CT/GG** | 1 | 1 |
| **TT/GG** | 0 | 1 |
| **TT/AG** | 1 | 2 |
| **TT/AA** | 1 | 1 |
| **c.665C>T *MTHFR* / c.66A>G *MTRR*** | | |
| **Combinations of genotypes** | **Study group** | **Control group** |
| **CC/AA** | 2 | 1 |
| **CC/AG** | 3 | 5 |
| **CC/GG** | 4 | 3 |
| **CT/AA** | 2 | 1 |
| **CT/AG** | 4 | 5 |
| **CT/GG** | 3 | 1 |
| **TT/GG** | x | x |
| **TT/AA** | 1 | 0 |
| **TT/AG** | 1 | 4 |
| **c.665C>T *MTHFR* / c.776G>C *TCN2*** | | |
| **Combinations of genotypes** | **Study group** | **Control group** |
| **CC/CC** | 1 | 2 |
| **CC/GC** | 4 | 5 |
| **CC/GG** | 4 | 2 |
| **CT/CC** | 2 | 5 |
| **CT/GC** | 6 | 2 |
| **CT/GG** | 1 | 0 |
| **TT/CC** | 0 | 2 |
| **TT/GC** | 2 | 2 |
| **TT/GG** | x | x |
| **c.66A>G *MTRR* / c.2756A>G *MTR*** | | |
| **Combinations of genotypes** | **Study group** | **Control group** |
| **AA/AA** | 3 | 2 |
| **AA/AG** | 2 | 0 |

| | | |
|---|---|---|
| **AA/GG** | x | x |
| **AG/AA** | 4 | 6 |
| **AG/AG** | 3 | 6 |
| **AG/GG** | 1 | 2 |
| **GG/AA** | 6 | 1 |
| **GG/AG** | 1 | 3 |
| **GG/GG** | | x |

| **c.665C>T *MTHFR* / c.66A>G *MTRR* / c.776G>C *TCN2*** | | |
|---|---|---|
| **Combinations of genotypes** | **Study group** | **Control group** |
| **CC/AA/GC** | 2 | 1 |
| **CC/AG/CC** | 1 | 0 |
| **CC/AG/GC** | 1 | 4 |
| **CC/AG/GG** | 1 | 1 |
| **CC/GG/CC** | 0 | 2 |
| **CC/GG/GC** | 1 | 0 |
| **CC/GG/GG** | 1 | 3 |
| **CT/AA/CC** | 1 | 1 |
| **CT/AA/GC** | 1 | 0 |
| **CT/AG/CC** | 0 | 4 |
| **CT/AG/GG** | 1 | 0 |
| **CT/GG/CC** | 1 | 0 |
| **CT/GG/GC** | 2 | 1 |
| **TT/AA/GC** | 1 | 0 |
| **TT/AG/CC** | 0 | 2 |
| **TT/AG/GC** | 1 | 2 |
| **CT/AG/GC** | 3 | 1 |
| **CC/AA/CC** | x | x |
| **CC/AA/GG** | x | x |
| **CT/AA/GG** | x | x |
| **CT/GG/GG** | x | x |
| **TT/AA/CC** | x | x |
| **TT/AA/GG** | x | x |
| **TT/AG/GG** | x | x |
| **TT/GG/CC** | x | x |
| **TT/GG/GC** | x | x |
| **TT/GG/GG** | x | x |

**Table S3.** Assessment of the relationship between the occurrence of alternative genotype variants **in both hetero- and homozygous** systems with the concentration of the tested biochemical parameters: **A)** in all patients and **B)** in the control group, based on multivariate regression model analysis. (The study group is in the main manuscript)

| **A) Parameters of all patients** | **c.1286A>C *MTHFR*** | | **c.665C>T *MTHFR*** | | **c.2756A>G *MTR*** | | **c.66A>G *MTRR*** | | **c.776G>C *TCN2*** | |
|---|---|---|---|---|---|---|---|---|---|---|
| | **AC+CC (n=18)** | | **CT+TT (n=22)** | | **AG+GG (n=18)** | | **AG+GG (n=33)** | | **GC+CC (n=33)** | |
| | **Coefficient** | **p-value** | **Coefficient** | **p-value** | **Coefficient** | **p-value** | **Coefficient** | **p-value** | **Coefficient** | **p-value** |
| **free ß-hCG_MoM** | 0.8502 | 0.3346 | 0.08496 | 0.9228 | -0.7974 | 0.2948 | -0.9825 | 0.3475 | 1.4238 | 0.1009 |
| **PAPP-A MoM** | -0.1609 | 0.4591 | 0.01353 | 0.9503 | -0.08778 | 0.6388 | 0.303 | 0.2434 | -0.2968 | 0.1645 |
| **MMA [ng/ml]** | -0.5401 | 0.9383 | 3.2207 | 0.6461 | -3.768 | 0.5324 | 2.2231 | 0.7785 | 0.93 | 0.8913 |
| **$Folate_{RBC}$ [ng/ml]** | -11.4334 | 0.6747 | -37.9641 | 0.17 | -6.2592 | 0.7896 | -43.9624 | 0.1593 | -10.5983 | 0.6896 |
| **$Folate_{SER}$ [ng/ml]** | 0.4033 | 0.9155 | -5.0571 | 0.1903 | -1.4483 | 0.6589 | 0.03471 | 0.9936 | 0.64 | 0.8628 |
| **HCY [μmol/L]** | -0.3904 | 0.6196 | -0.1453 | 0.8536 | 0.9109 | 0.1839 | -0.1358 | 0.8784 | -0.03966 | 0.9586 |
| **Vit. B12 [pg/ml]** | 10.6455 | 0.0796 | 8.5442 | 0.1572 | 1.4 | 0.7844 | 5.3863 | 0.4245 | 2.477 | 0.6687 |

| **B) Parameters of the control group** | **MTHFR c.1286A>C** | | **MTHFR c.665C>T** | | **MTR c.2756A>G** | | **MTRR c.66A>G** | | **TCN2 c.776G>C** | |
|---|---|---|---|---|---|---|---|---|---|---|
| | **AC+CC (n=11)** | | **CT+TT (n=11)** | | **AG+GG (n=11)** | | **AG+GG (n=18)** | | **GC+CC (n=18)** | |
| | **Coefficient** | **p-value** | **Coefficient** | **p-value** | **Coefficient** | **p-value** | **Coefficient** | **p-value** | **Coefficient** | **p-value** |
| **free ß-hCG_MoM** | 0.198 | 0.6248 | 0.2614 | 0.5335 | 0.04537 | 0.8991 | -0.1894 | 0.7334 | 0.3242 | 0.5706 |
| **PAPP-A MoM** | -0.5744 | 0.0648 | -0.2348 | 0.4413 | -0.1953 | 0.4554 | 0.1942 | 0.6302 | 0.2341 | 0.5714 |
| **MMA [ng/ml]** | 6.1495 | 0.2947 | -6.4185 | 0.2903 | -1.0819 | 0.8323 | -11.136 | 0.1741 | 8.9111 | 0.2819 |
| **$Folate_{RBC}$ [ng/ml]** | 20.8694 | 0.4587 | 16.1544 | 0.5774 | 2.2524 | 0.9275 | 22.9762 | 0.552 | -65.7639 | 0.1105 |
| **$Folate_{SER}$ [ng/ml]** | 6.0968 | 0.123 | 0.6711 | 0.8638 | 4.156 | 0.228 | -6.9579 | 0.195 | 4.396 | 0.4152 |
| **HCY [μmol/L]** | -0.6598 | 0.4687 | -0.2139 | 0.8188 | 0.481 | 0.5505 | 0.8337 | 0.5054 | -0.1033 | 0.9352 |
| **Vit. B12 [pg/ml]** | 13.7762 | 0.1726 | 3.1281 | 0.7569 | -6.4541 | 0.4606 | 1.5957 | 0.9054 | -2.5343 | 0.8539 |

free β-hCG MoM: median of the free β-hCG subunit; PAPP-A MoM: median PAPP-A; MMA: methylmalonic acid; $Folate_{RBC}$: red blood cell folate; $Folate_{SER}$: serum folate; HCY: homocysteine; Vit.B12: Vitamin $B_{12}$.

**Table S4.** Analysis of the correlation between the concentrations of the biochemical parameters and the age of the patients qualified for the study based on Spearman's correlation coefficient.

| Parameters | Patient age | |
|---|---|---|
| | Coefficient | p-value |
| **HCY [μmol/L]** | -0.104 | 0.5143 |
| **Vit.$B_{12}$ [pg/ml]** | 0.123 | 0.4420 |
| **$Folate_{RBC}$ [ng/ml]** | -0.0977 | 0.5419 |
| **$Folate_{SER}$ [ng/ml]** | -0.0711 | 0.6803 |
| **MMA [ng/ml]** | 0.0708 | 0.6583 |
| **PAPP-A MoM** | 0.176 | 0.2776 |
| **free β-hCG MoM** | -0.164 | 0.3126 |

HCY: homocysteine; Vit.B12: Vitamin $B_{12}$; Folate $_{RBC}$: red blood cell folate, Folate $_{SER}$: serum folate; MMA: methylmalonic acid; PAPP-A MoM: median PAPP-A; free β-hCG MoM: median of the free β-hCG subunit.
